# Role of TRPV1 ion channel in cervical squamous cell carcinoma genesis

**DOI:** 10.3389/fmolb.2022.980262

**Published:** 2022-08-22

**Authors:** Zhenming Wang, Junhong Dong, Wenxiu Tian, Sen Qiao, Hongmei Wang

**Affiliations:** ^1^ Weifang People’s Hospital, Weifang, China; ^2^ School of Basic Medicine, Weifang Medical University, Weifang, China; ^3^ Central of Translation Medicine, Zibo Central Hospital, Zibo, China; ^4^ Department of Pharmacology, Center for Molecular Signaling (PZMS), Saarland University School of Medicine, Homburg, Germany; ^5^ Assisted Reproduction Center, Northwest Women's and Children's Hospital, Xi'an, China; ^6^ Department of Pharmacology, School of Medicine, Southeast University, Nanjing, China

**Keywords:** cervical squamous cell carcinoma, TRPV1, inflammatory response, methylation, ferroptosis

## Abstract

The transient receptor potential (TRP) family is a widely expressed superfamily of ion channels that regulate intracellular Ca^2+^ homeostasis and signal transduction. Abnormal expression of TRPV1 is closely related to malignant tumors of the female reproductive system such as breast, ovarian, cervical and endometrial cancers. In this study, we found a significant reduction of TRPV1 expression in cervical squamous cell carcinoma and this expression is inversely association with the risk of cervical squamous cell carcinoma. Furthermore, TRPV1 is involved in cell differentiation, iron death, inflammatory response, and metabolic regulation in cervical squamous cell carcinoma. Meanwhile TRPV1 is positively correlated with T cells and negatively associated with macrophages, indicating that TRPV is associated with tumor cell immunity. Therefore, TRPV1 may be a potential marker of cervical cancer and a promising anti-cancer drug candidate.

## Introduction

Cervical cancer is the second most common female malignant tumor in the world, including squamous cell carcinoma of cervix and cervical adenocarcinoma, accounting for the fourth leading cause of death in female cancer patients. The annual mortality and new cases of cervical cancer are about 275/500,000 worldwide (Olusola et al., 2019). With the development of cervical cancer vaccine and the universal promotion of cervical cancer screening, the incidence and mortality of cervical cancer are significantly lower than before, but the incidence rate shows a younger development trend, which seriously threatens the health and quality of life of young women (Buskwofie et al., 2020). Therefore, it is urgent to clarify the etiology of cervical cancer and explore its prevention mechanism, which will help to reduce the incidence of cervical cancer and improve its prognosis.

TRPs channel is a kind of non-selective cation channel located on the cell membrane, which plays a role by influencing downstream calcium signal molecules. It contains seven subfamilies: TRPC (Cano-nical), TRPV (Vaniloid), TRPM (Melastatin), TRPML (Mucolipin), TRPA (Ankyrin), TRPP (Polycystin), and TRPN (nomp C-like). Recent studies have shown that TRPC, TRPM, and TRPV family are closely related to the proliferation, invasion, and metastasis of malignant tumors. They are not only involved in the occurrence and development of GBM, skin cancer, liver cancer, colon cancer, gastric cancer, but also closely related to the occurrence of female reproductive system malignant tumors (Liberati et al., 2013; Jimenez et al., 2020; Stewart, 2020; Lefranc, 2021). Inhibition of TRPV6 expression decreased the influx of Ca^2+^, and decreased the ability of proliferation of breast cancer (Erin, 2020). The abnormal expression of TRPV1 is related to the survival period of breast cancer patients (So et al., 2020). Inhibition of TRPM3 or TRPV6 expression can significantly reduce the growth of ovarian tumor (Li et al., 2020a). The expression level of TRPM7 was positively correlated with pelvic lymph node metastasis and poor prognosis of ovarian cancer (Liu et al., 2019). However, inhibition of TRPV1 expression promoted the proliferation of ovarian cancer cells (Han et al., 2020). This indicates that different members of TRP family play different roles in tumorigenesis and development. Studies have found that inhibition of TRPC6 expression can block endometrial cancer cells in G2/M phase (Van den Eynde et al., 2021). TRPV4 is also involved in the migration of endometrial cancer cells (Li et al., 2020b). TRPM4 is negatively correlated with the proliferation of cervical cancer HeLa cells. TRPM4 blockers can significantly inhibit the growth of HeLa cells and arrest the cell cycle in G0/G1 phase (Borgstrom et al., 2021). The increased expression of TRPC6 may be involved in the progression of cervical cancer (Bai et al., 2022). TRPV subfamily is widely involved in the process of malignant tumors of female reproductive system, but the mechanism is not clear.

Chronic inflammation has long been considered as an important cause of cancer. Inflammatory response in tumor microenvironment is an important part of tumor formation and development, and also an important marker of tumor formation. Cervical cancer mainly occurs in the cervical epithelial transition area, which is the most common area of cervicitis. Some studies have found that inflammation is closely related to the proliferation, invasion, metastasis and prognosis of cervical cancer cells. The level of IL-8 in cervical cancer tissue was significantly higher than that in normal cervical tissue. In recent years, studies have found that TRPV1 plays a protective role in some inflammation. In endotoxic shock model, TRPV1 gene deletion leads to increased risk of septic shock by increasing intra-abdominal inflammatory mediators and liver damage. According to Clark et al. (2007), in the LPS induced abdominal sepsis model, the incidence of hypotension shock and hypothermia in TRPV1 gene knockout mice was significantly higher than that in the wild control group. At the same time, the levels of TNF-a and N0 in peritoneal exudate were also significantly increased. These results suggest that TRPV1 gene knockout accelerates the inflammatory process, which further indicates that TRPV1 may play a protective role in the inflammatory process. However, it is not clear whether TRPV1 is involved in the progression of cervical cancer through immune inflammatory response.

The expression of TRPV1 in human cervical cancer cell lines and tissues has been reported. This study found that the expression of TRPV1 in cervical cancer, especially in cervical squamous cell carcinoma, was significantly decreased. However, the specific mechanism of TRPV1 down-regulation in the progression of cervical cancer and its impact on the prognosis of patients with cervical cancer need to be further explored. Therefore, we used bioinformatics database to search for the possible mechanism of inducing the proliferation and metastasis of cervical cancer, and to explore the effect of TRPV1 down-regulation on the prognosis of patients with cervical cancer, so as to provide a possible target for the diagnosis and treatment of cervical cancer.

## Materials and methods

### TCGA

TCGA is a publicly available database, covering cervical squamous cell carcinoma (*n* = 253), endocervical adenocarcinoma (*n* = 52). And normal patients (*n* = 19). We used TCGA database to analyze the TRP expression level in cervical cancer.

### GEPIA

GEPIA (http://gepia.cancer-pku.cn/index.html) is a developed interactive web server for analyzing the RNA sequencing expression data. In this study, we performed the multiple gene comparison analysis of TRP channel using the “cervical squamous cell carcinoma” dataset.

### Kaplan–Meier Plotter

Kaplan–Meier Plotter (https://kmplot.com/analysis/) is a useful prognostic biomarker assessment tool that explores the effect of TRPs on survival in CSCC using the databases from TCGA. To analyze the prognostic value of TRP channel in CSCC regarding overall survival (OS) and disease specific survival (DSS), the patient samples were split into two groups by the median expression, with the restricted analysis to subtype histology.

### cBioPortal

cBioPortal (www.cbioportal.org) is a comprehensive web resource that could visualize and analyze multidimensional cancer genomics data. Based on the TCGA database, genetic alterations of TRP channel were obtained from cBioPortal, including 305 cervical cancer samples (TCGA, PanCancer Atlas). We used this website to analyze the genetic mutation of TRP family.

### Bioinformatics

Bioinformatics (https://bioinformatics.com.cn) and (https://www.home-for-researchers.com) are the data analysis and bioinformatics online analysis website capable of a scientific drawing. In this study, we used this website to perform protein correlation analysis of TRP family.

### Quantitative real time polymerase chain reaction

The total RNA from normal tissues and CSCC tissues was extracted with TRIzol^®^ reagent (Thermo Fisher Scientific, United States). The total RNA was then reverse transcripted into cDNA by using the PrimeScript™ RT Master Mix kit (United States) according to the instructions. qPCR reactions were performed with the SYBR^®^ Premix Ex TaqTM II kit kit (Takara Biotechnology, United States) with specific primer sequences with the annealing temperature at 60°C. The forward primer of TRPV1 was 5′-AGC​GTT​TGT​CGA​CTG​ACT​GA-3′ and the reverse primer was 5′-CCT​TTT​CCT​CTG​ACG​GGT​CC-3′. The forward primer of b-actin was 5′ -CATGGG CCAGAAGgACTC-3′, and the reverse primer was 5′-AA GGT​CTG​GAG​CCA​GAT​C-3′. The relative expression is compared with by calculating (2^−ΔΔCt^) values.

### Western blotting

After the extraction of normal tissue and CSCC tissue total protein, SDS-PAGA electrophoresis was performed, and the membrane was incubated in 5% skimmed milk powder and blocked for 1 h at room temperature. The membrane was then incubated with either Murine anti- TRPV1 (1:1000, 66983-1, Wuhan Sanying Biotechnology Co., Ltd., China) and Murine anti-β-actin (1:1000, 66009-1, Wuhan Sanying Biotechnology Co., Ltd., China) overnight at 4°C. The secondary antibody was incubated with HRP-labeled sheep anti-rabbit antibody (1:3000) or sheep anti-murine antibody (1:10000) at room temperature for 1.5 h. ECL substrate was then used for detection. Absorbance (A) values of protein bands was analyzed.

### Immunohistochemistry

Normal tissue and CSCC tissue were embedded with paraffin. These tissued were then sectioned with 4 μm thickness. Slices were incubated at 60°C for 2 h, then were incubated in 3% hydrogen peroxide solution for 10 min, followed by PBS washing, then the slices were incubated in boiling EDTA repair solution for 20 min for antigen retrieval, followed by another wash PBS washing. Slices were then incubated with 5% bovine serum albumin blocked for 20 min, followed by incubation with TRPV1 antibody overnight at 4°C. The slices were then incubated with secondary antibody for 1.5 h. Finally, the expression of TRPV1 was analyzed after DAB and hematoxylin staining.

### Statistical analysis

We downloaded CSCC RNA-seq data from The Cancer Genome Atlas (TCGA) database and Gene Expression Omnibus (GEO) as well as pairing mRNA expression data from normal tissue samples. All human tissue studies were performed with the approval from the Affiliated Hospital of Weifang Medical College Review Board with informed consent obtained from patients. Tissues of CSCC cancer were obtained from a total of five patients. Statistical analysis was performed by using GraphPad 5.0 software (United States). The student t test was used to compare significant differences between two groups. The survival curve is plotted using the Kaplan-Meier method and compared with the log-rank test. All values are expressed as mean ± standard deviation (SD). RT-qPCR, western blotting and Immunohistochemistry were repeated at least three times.

## Results

### Altered expression of TRPs has been associated with CSCC

**FIGURE 1:**
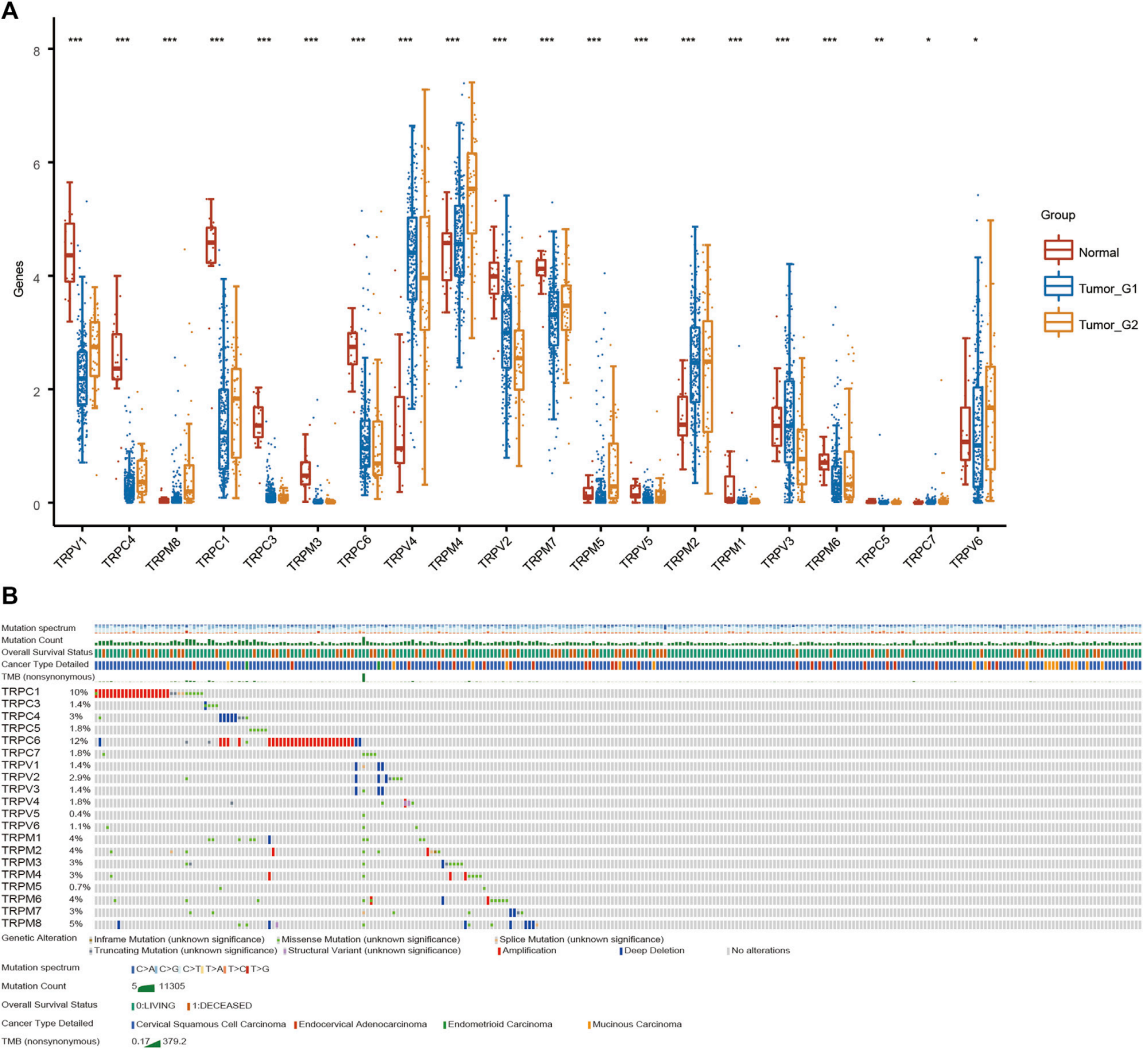
Analysis of TRPs expression alterations in cervical cancer. **(A)** Expression analysis of TRP family in cervical cancer. **(B)** Mutations of TRPs in cervical cancer.

To explore the role of TRPs in cervical cancer, the expression levels of TRPs were analyzed in cervical squamous cell carcinoma (CSCC) and cervical adenocarcinoma with TCGA database, and we found that TRPs were differently expressed in cervical cancer. The expression levels of TRPV (Olusola et al., 2019; Buskwofie et al., 2020), TRPC (Liberati et al., 2013; Olusola et al., 2019; Jimenez et al., 2020; Lefranc, 2021) and TRPM (Liberati et al., 2013; Olusola et al., 2019; Erin, 2020; Jimenez et al., 2020; Stewart, 2020) were decreased in cervical squamous cell carcinoma and cervical adenocarcinoma, with more obvious trend of TRPV1 and TRPC1, while the expression of TRPV (Stewart, 2020; Lefranc, 2021) and TRPM (Buskwofie et al., 2020; So et al., 2020) increased (Figure 1A). Interestingly, TRPM4 showed higher expression only in cervical adenocarcinoma; while TRPV3 showed lower expression only in cervical adenocarcinoma. TRPM4 and TRPV3 showed no significantly difference between normal group and CSCC. TRPM4 silencing was reported to promote GSK-3β-dependent degradation of β-catenin and reduce β-catenin/Tcf/Lef-dependent transcription to inhibit cervical cancer (Armisen et al., 2011). TRPV6 was expressed in lower level in CSCC and higher in cervical adenocarcinoma compared to normal group. This indicated that TRPs may participate in the regulation of different mechanisms both CSCC and cervical adenocarcinoma. Then, we analyzed the alterations of TRPs in CSCC and cervical adenocarcinoma. The TRPC and TRPM families have relatively high mutation rates (Figure 1B). TRPC1 and TRPC6 mutations mainly occurred in cervical adenocarcinoma, while TRPC4, TRPV (Liberati et al., 2013; Olusola et al., 2019; Buskwofie et al., 2020) and TRPM (Erin, 2020; So et al., 2020) were mainly mutated in cervical squamous cell carcinoma, which indicated that TRPs mutations could be one of the causes of cervical cancer. Some research reported that mutations in TRP channel genes result in abnormal regulation of TRP channel function or expression, and interfere with normal spatial and temporal patterns of intracellular local Ca^2+^ distribution (Yang and Kim, 2020). This has important implications for the research of familial cervical cancer.

### TRPs are associated with survival and prognosis of CSCC

**FIGURE 2:**
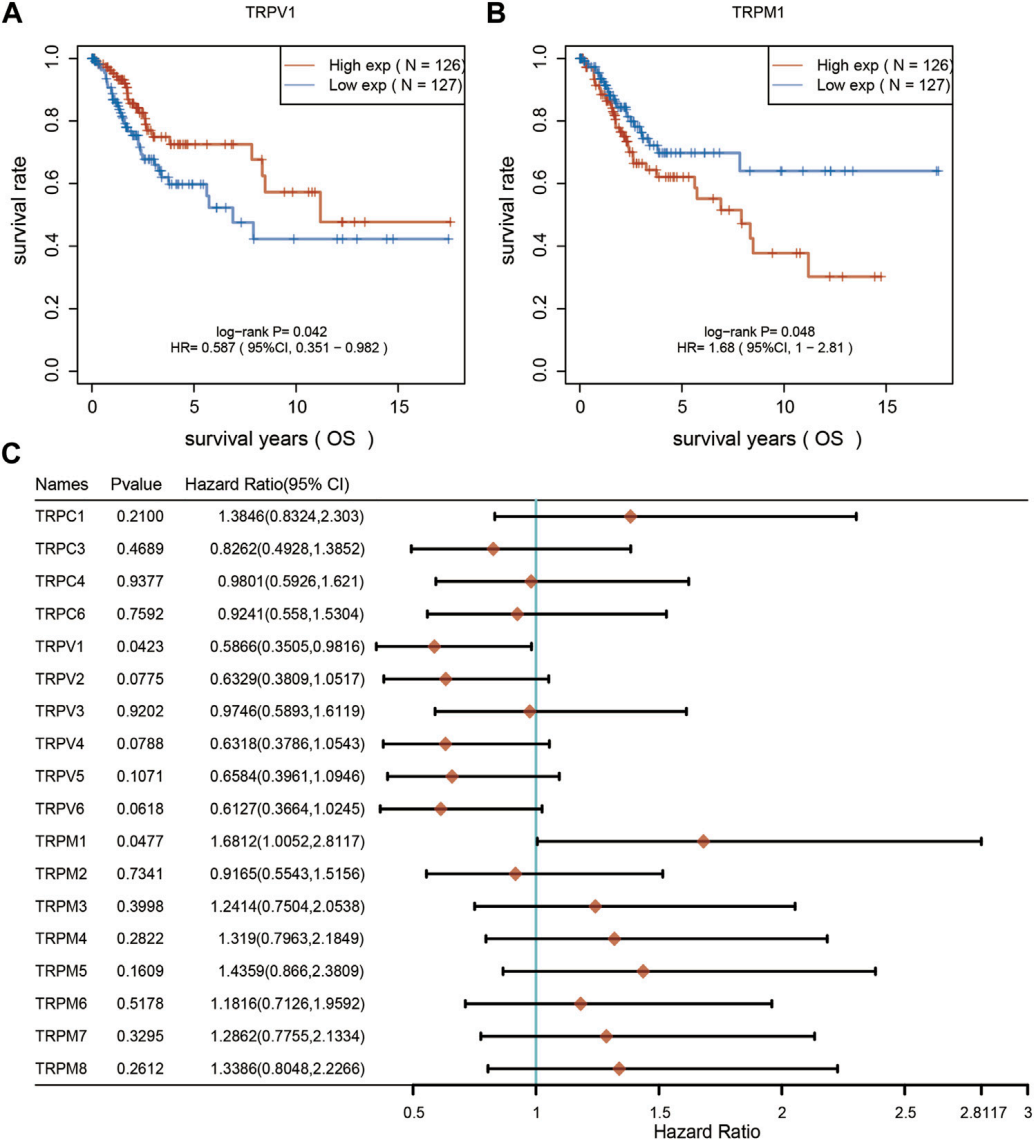
Overall survival curves were used to evaluate the prognosis of CSCC patients. **(A,B)** From left to right, overall survival analysis based on TRPV1 and TRPM1 expression. **(C)** Overall survival curves of TRPs were analyzed in CSCC patients.

**FIGURE 3:**
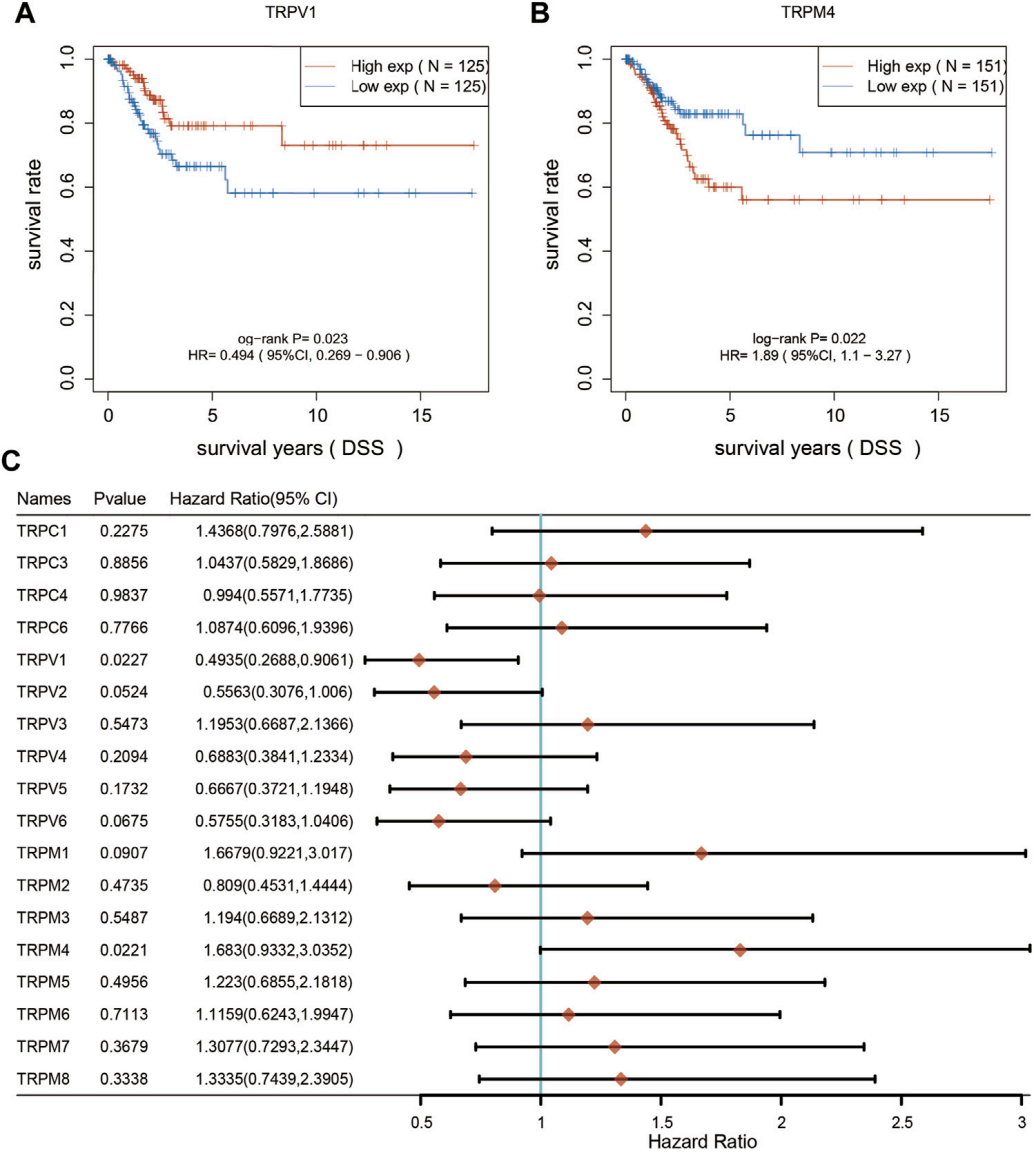
Disease specific survival curves were used to evaluate the prognosis of CSCC patients. **(A,B)** From left to right, DSS analysis based on TRPV1 and TRPM4 expression. **(C)** DSS curves of TRPs were analyzed in CSCC patients.

Furthermore, we analyzed the overall survival (OS) and disease specific survival (DSS) data of cervical cancer, and found that the overall survival prognosis of patients with low expression of TRPV1 showed worse prognosis, while patients with high expression of TRPM1 showed worse prognosis (Figures 2A,B). On DSS, lower TRPV1 level presented worse prognosis, while higher TRPM4 level presented worse prognosis in CSCC (Figures 3A,B). Then the correlation between TRPs and hazard ratio (HR) was detected, and found that the TRPV1 was negatively correlated with cancer risk; TRPM1 and TRPM4 were positively correlated with cancer risk (Figures 2C, 3C).

### Functional analysis of TRPs in CSCC

**FIGURE 4:**
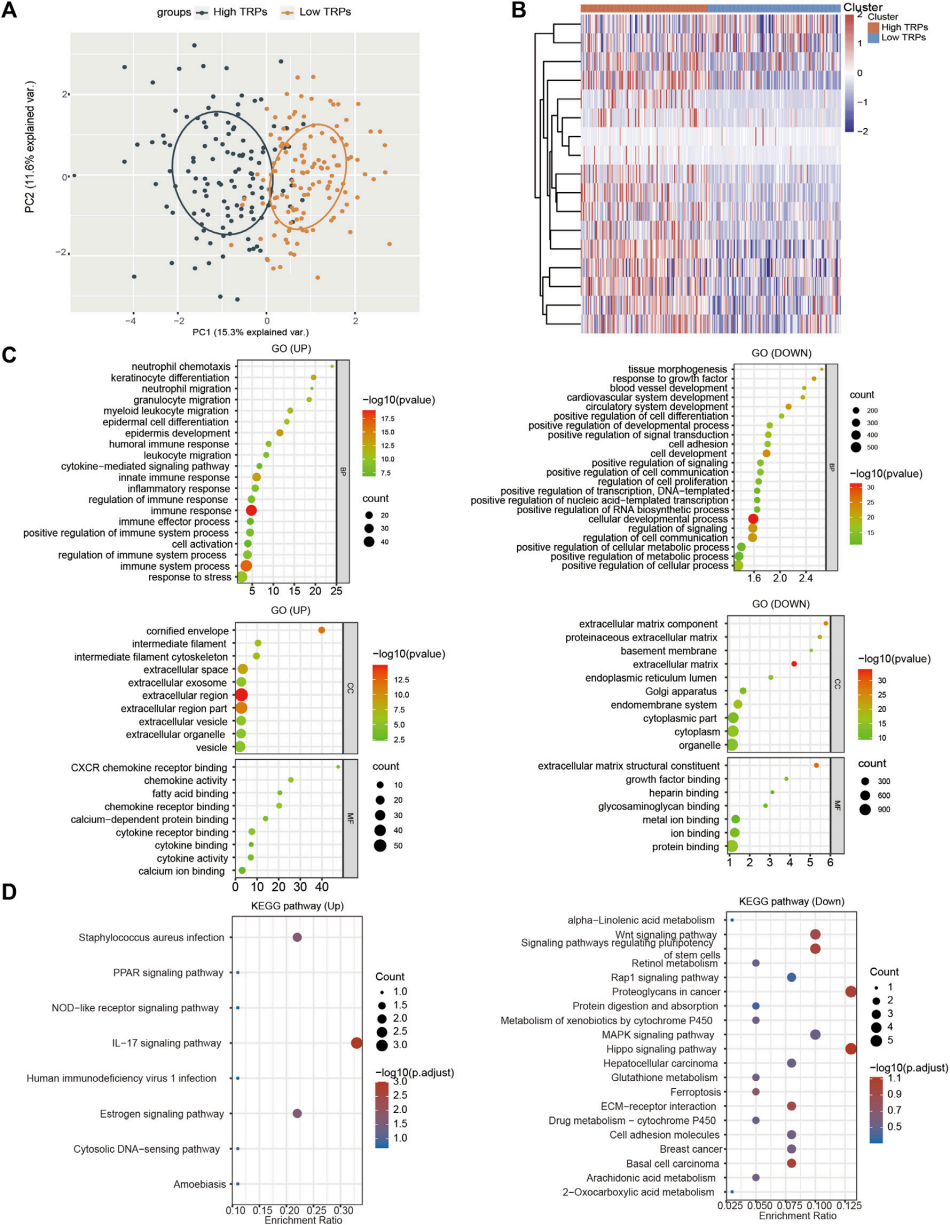
Functional analysis of TRPs family in CSCC. **(A)** PCA analysis based on the expression levels of TRPs. **(B)** Heatmap show the high expressed TRPs and low expressed TRPs in two clusters. **(C,D)** The GO analysis and KEGG (Kyoto Encyclopedia of Genes and Genomes) analysis were performed on TRPs, and the correlation was evaluated with the enrichment score.

According to the differential expression analysis of TRPs and the effect of multiple survival time in CSCC, we divided CSCC into low expression group and high expression group of TRPs for functional comparison (Figures 4A,B). We found that most genes (*n* = 1423) were down-regulated by low TRPS levels, and some genes (*n* = 91) were up-regulated in low expression group compared to high expression group (FC > 1.5, *p* < 0.05). For up-regulated genes, KRT family and S100 family were reported that they were related to cell proliferation, cell apoptosis, metastasis, radiation resistance characteristics in many cancers. For instance, enhanced KRT13 gene expression bestows radiation resistance in squamous cell carcinoma cells (Nguyen et al., 2021). KRT13 also promotes stemness and drives metastasis in breast cancer through a plakoglobin/c-Myc signaling pathway (Yin et al., 2022). KRT6B was considered as a key mediator of notch signaling in honokiol-induced human hepatoma cell apoptosis (Zhang et al., 2015). High expression of keratin 6C is associated with poor prognosis and accelerates cancer proliferation and migration by modulating epithelial-mesenchymal transition in lung adenocarcinoma. The elevated level of KRT6C was related to worse prognosis in LUAD patients (Hu et al., 2020). Calprotectin (S100A8/S100A9), a heterodimeric EF-hand Ca^2+^ binding protein, are abundant in cytosol of neutrophils and are involved in inflammatory processes and several cancerous pathogens (Shabani et al., 2018). For down-regulated genes by TRPs, ALDH1A1 modulates the activity of lysosomal autophagy inhibitors in cancer cells (Piao et al., 2017). ALDH1A1 was also involved in ferroptosis of gastric cancer (Ni et al., 2022). Aberrant promoter methylation of EFHD1 was highly positive in colorectal cancer plasma samples, and they might be useful in detection of early-stage colorectal cancer (Takane et al., 2014). Hypermethylation was identified near CLIC6 encoding predominantly transcription factors in human salivary gland adenoid cystic carcinoma (Bell et al., 2011).

To further confirm the functional analysis between low expression group and high expression group of TRPs, we utilized Gene Ontology (GO) enrichment analysis to identify specific biological processes (BP). Analysis of low expression group vs. high expression group of TRPs revealed that the regulated genes were significantly enriched in sequestering of metal ion, immune response, cell differentiation, and so on (Figure 4C). For KEGG analysis, they primarily contained signaling pathways associated with PPAR signaling pathway, IL17 signaling pathway, and estrogen signaling pathway; down-regulated pathways like Wnt signaling pathway, MAPK signaling pathway, and Ferroptosis (Figure 4D). Based on the analysis, we found that the low expression TRPs could lead to inflammatory response, ferroptosis, and cell differentiation. We speculated that low-expressed TRPs, especially TRPV1, play a key role in the progression of CSCC through regulating inflammatory response, ferroptosis, and cell differentiation.

### TRPV1 is involved in multiple pathways regulating CSCC progression

**FIGURE 5:**
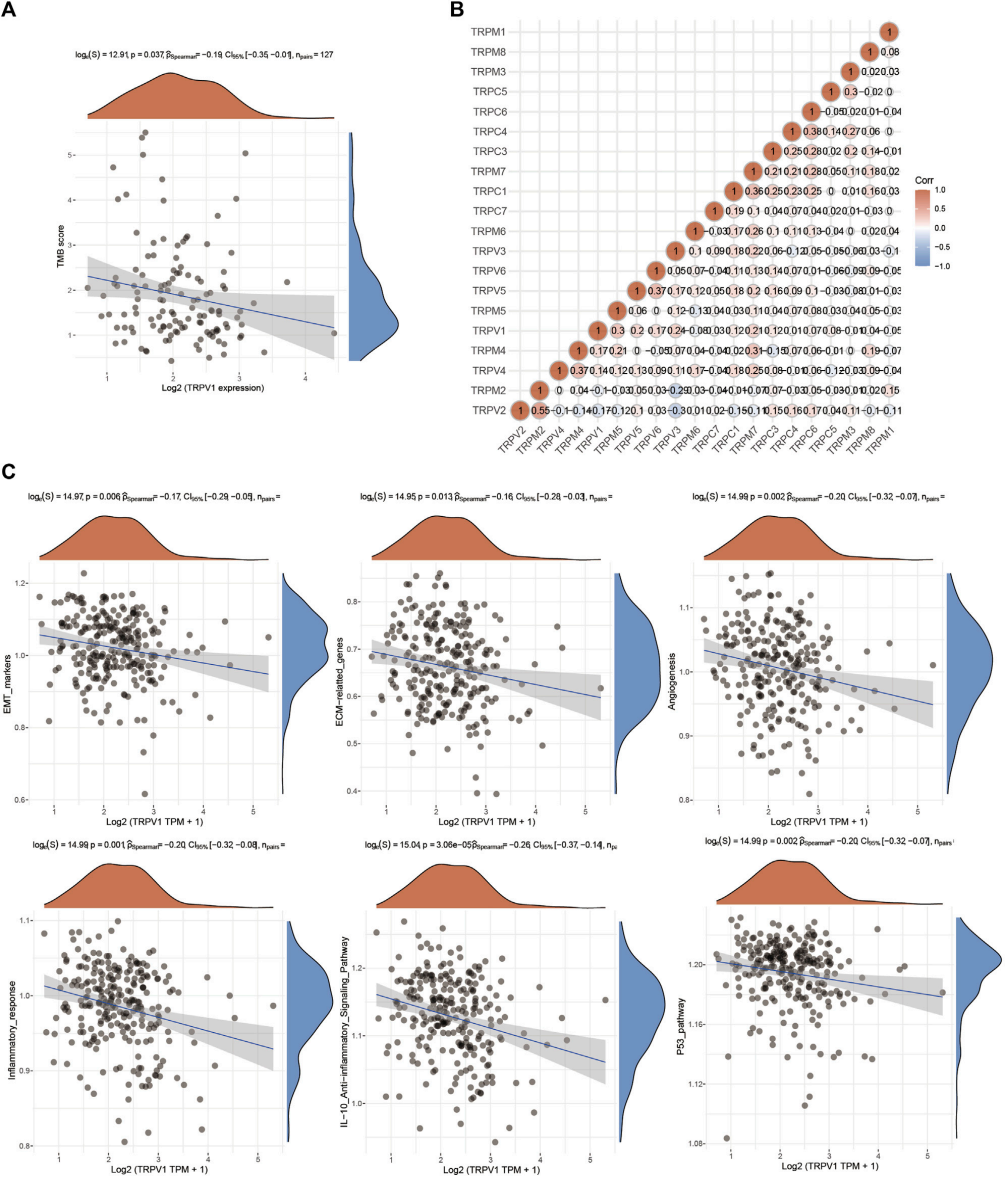
The function of TRPV1 in CSCC. **(A)** Correlation analysis between TRPV1 expression and TMB was performed using Spearmans method. The abscissa represents gene expression distribution, and the ordinate represents TMB score distribution. The density curve on the right represents the distribution trend of TMB score; the upper density curve represents the distribution trend of gene expression. The values on the top represents the correlation *p* value, correlation coefficient and correlation calculation method. **(B)** Gene correlation analysis of TRPs in CSCC. **(C)** The regulatory mechanism of TRPV1 on signaling pathways.

TRPV1 affected the prognosis of CSCC. Tumor mutational burden (TMB) is of interest in immunotherapy; TMB and PDL1 is two important biomarkers for predicting response to PD1 antibody therapy. Furthermore, the correlation of TRPV1 and TMB was analyzed in CSCC (Figure 5A). Low expressed TRPV1 showed negative correlation to TMB. Through gene correlation analysis, we found a strong correlation between TRPV (Olusola et al., 2019; Buskwofie et al., 2020; Lefranc, 2021) and TRPM (Buskwofie et al., 2020; Stewart, 2020; Lefranc, 2021) (Cor > 0.3) (Figure 5B). This means TRPV1 could form heterotetramers to drive ion channel function in CSCC. TRPV1 affects extracellular matrix (ECM), epithelial-mesenchymal transition EMT (EMT), tumor angiogenesis, inflammation, and P53 pathways in the regulation of CSCC (Figure 5C). EMT confers cancer cells the ability of invasion and metastasis by regulating tumor immunologic escape. There may be interaction between TRPV1 and TRPM5. Further analysis revealed that TRPV2 and TRPM2, TRPV4 and TRPM4, and TRPV5 and TRPV6 may also had the interaction, which indicates that the role of TRPVs and TRPMs in the development of CSCC may be the result of the joint action of multiple subunits, which also provides ideas for studying the mechanism of action of TRPV1 and TRPM5 in CSCC.

### TRPV1 participates in inflammatory regulation in CSCC

**FIGURE 6:**
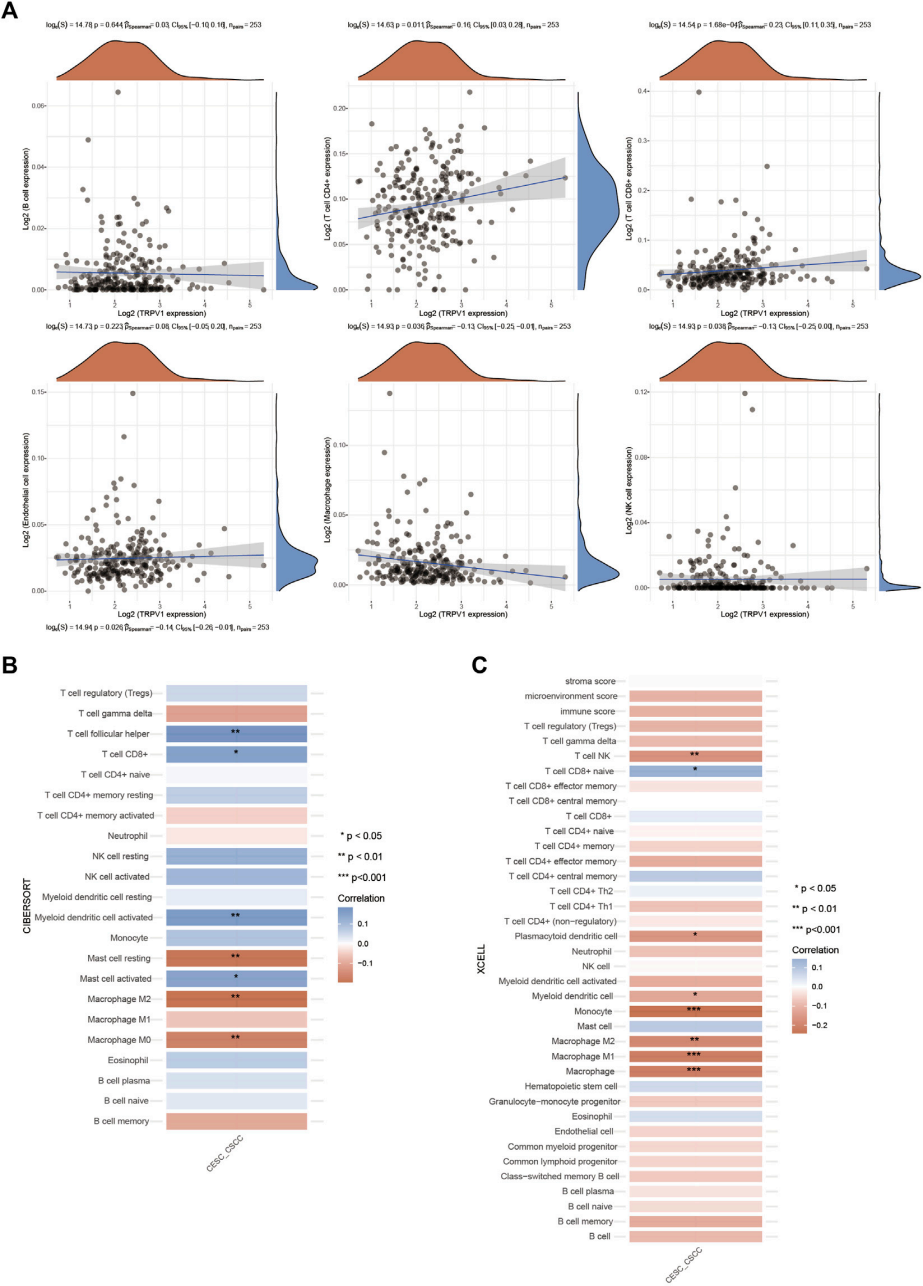
Regulation of TRPV1 on immune infiltrating cells in CSCC. **(A)** The correlations between TRPV1 expression and immune score was analyzed with Spearman. The abscissa represents the distribution of the gene expression or the score, and the ordinate represents the distribution of the immune score. The density curve on the right represents the trend in distribution of the immune score, the upper density curve represents the trend in distribution of the gene expression or the score. The value on the top represents the correlation *p* value, correlation coefficient and correlation calculation method. **(B,C)** The correlations among TRPV1 with CIBERSORT and XCELL methods: A heatmap of the correlation between TRPV1 and immune score. ** for *p* < 0.01, * for *p* < 0.05.

The regulation of inflammatory response is closely related to the development of tumor. We analyzed the correlation between TRPV1 and immune cells in cervical squamous cell carcinoma, and found that TRPV1 was positively correlated with T cell immune infiltration, and negatively correlated with macrophages and NK cells (Figure 6A). Then we further analyzed the correlation between TRPV1 and different subtypes of immune infiltration cells by different analysis methods (CIBERSORT and XCELL). TRPV1 was positively correlated with CD8^+^T cells and negatively correlated with macrophage M2 and NK cells (Figures 6B,C). The results suggest that TRPV1 is involved in the process of tumor immune regulation in CSCC. We speculate that TRPV1 may participate in tumor immunologic escape through regulating the infiltration of immune cells, and thus participate in the occurrence of cervical squamous cell carcinoma.

**FIGURE 7:**
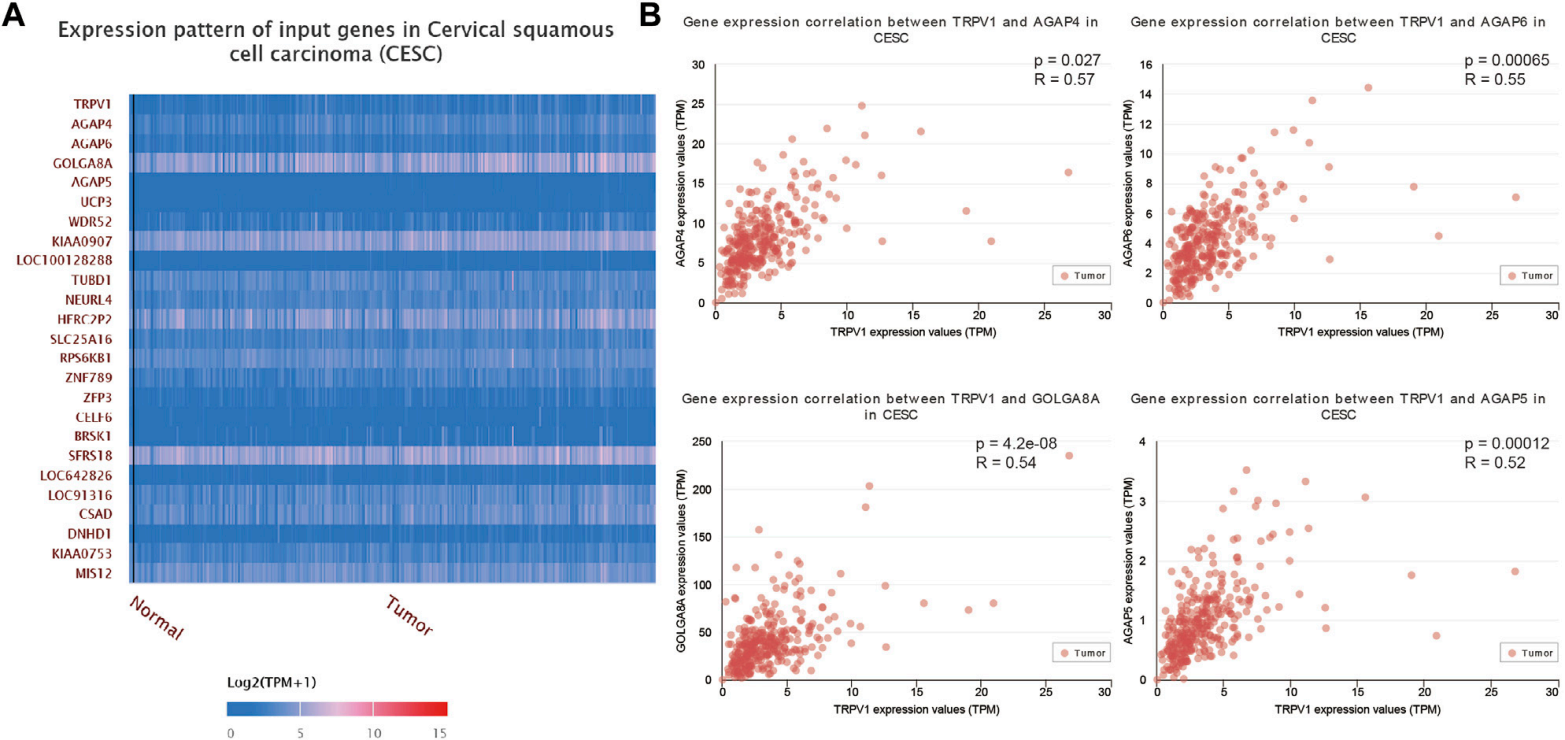
The correlation analysis of TRPV1 to other genes in CSCC. **(A)** The heatmap showed the 25 tops genes which were positively related to TRPV1. **(B)** AGAP4/5/6 and GOLGA8A showed positively correlation with TRPV1.

Gene expression alterations (top 25) were determined in CSCC (Figure 7A). TRPV1 was positively correlated with AGAP4, AGAP6, GOLGA8A, AGAP5, and other genes (Figures 7A,B). Most genes that were regulated by TRPV1 were located in subcellular organelles. GOLGA8A was expressed in Golgi, AGAP4/5/6 were related to metal ion binding and GTPase activator activity. AGAP4/6 expression alterations were proved that would result to multiple cutaneous basal cell carcinomas (Becker et al., 2017). That means TRPV1 is involved in the regulation of multiple mechanisms in the occurrence of cervical squamous cell carcinoma.

### The assessment of TRPV1 methylation level in CSCC

**FIGURE 8:**
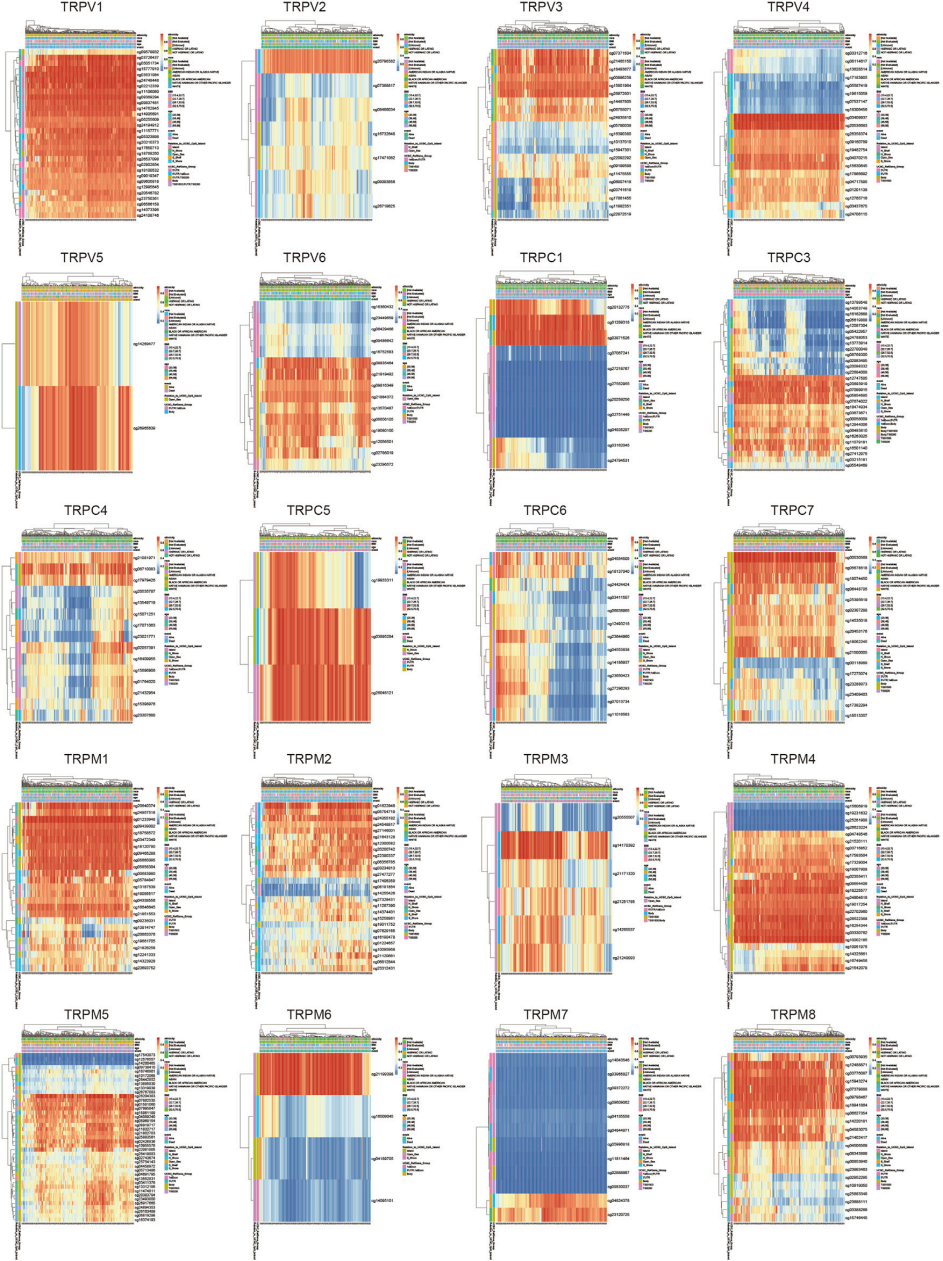
The prognostic value of the DNA methylation of TRPs signature in CSCC.

**FIGURE 9:**
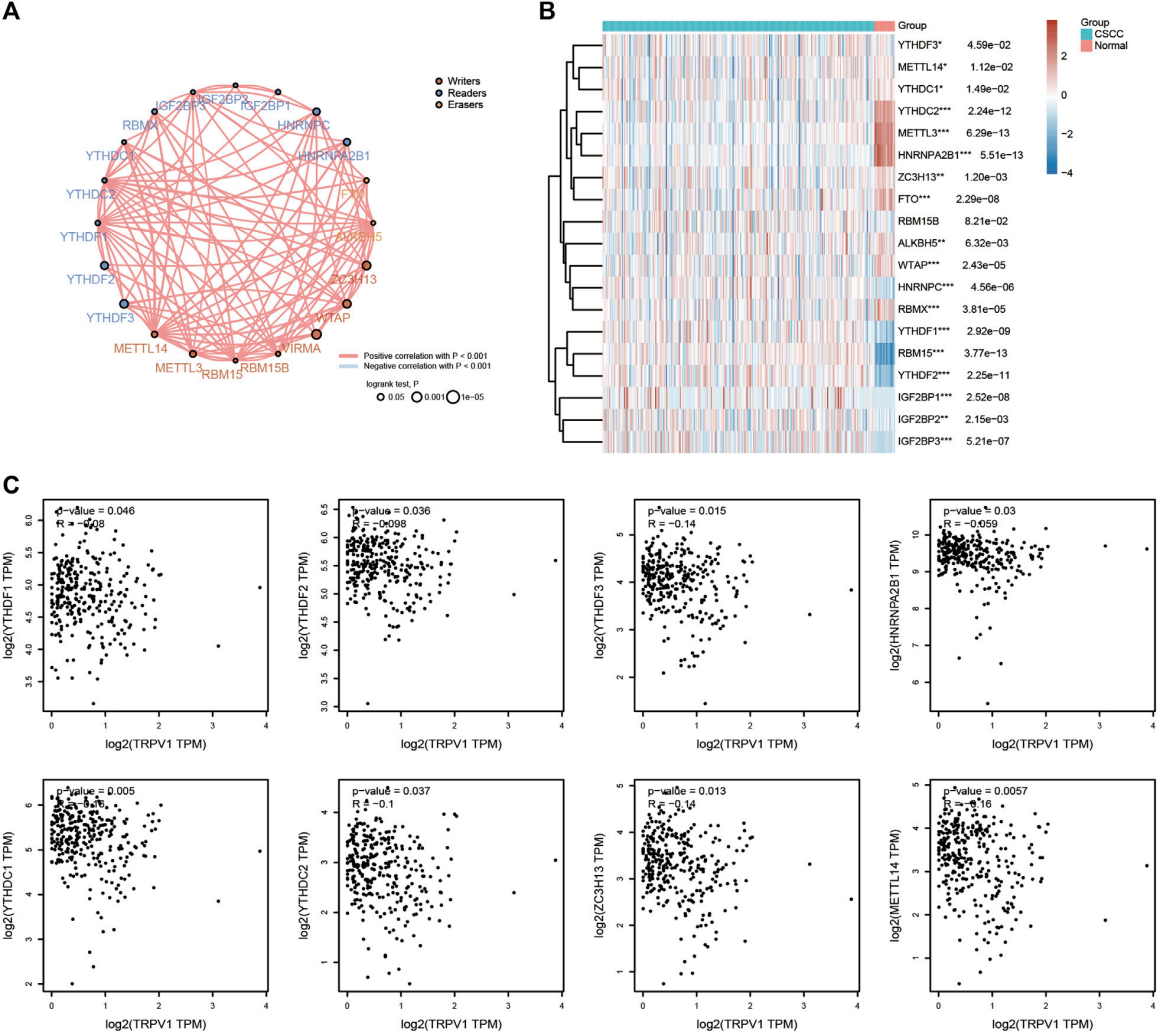
Effects of TRPV1 on methylases. **(A)** Circles represent the m6A-related mRNA, line represents the relationship between genes. Red represents positive correlation whereas blue represents negative correlation, the thicker the line, the higher the correlation coefficient. The larger the circle the smaller log rank p. Different colors of circles represent writers, readers and erasers. **(B)** The heatmap of m6A related gene expression. The different colors represent the trend of gene expression in different samples. *p < 0.05, **p < 0.01, ***p < 0.001, asterisks (*) stand for significance levels. The statistical difference of two groups was compared through the Wilcox test, significance difference of three groups was tested with Kruskal–Wallis test. **(C)** Effects of TRPV1 on methylases including YTHDF1/2/3, HNRNPA2B1, YTHDC1/2, ZC3H13, and METTL14.

Methylation can regulate gene expression by causing DNA conformation changes. We analyzed the methylation levels of different sites of TRPs, and found that TRPV1 showed the highest methylation levels among TRPs in CSCC. TRPM1 and TRPM4 also have more methylation sites in CSCC, except TRPV1 (Figure 8). Furthermore, it was found that the regulation of methylase by TRPV1 in USCC. RNA methylation is regulated by different types of regulators, including methyltransferases (“writers”), RNA-binding proteins (“readers”), and demethylases (“erasers”). These regulators showed positive correlation in CSCC (Figure 9A). Differential expression analysis revealed the regulators were up or down regulated in CSCC by heatmap (Figure 9B). Especially, TRPV1 negatively regulated YTHDF1/2/3, HNRNPA2B1, YTHDC1/2, ZC3H13, and METTL14 in CSCC (Figure 9C). In addition, we found that TRPV1 is not correlated with demethylase such as FTO, ALKBH5 (Supplementary Figure S2). In summary, it is tempting to speculate that the TRPV1 is correlated with methylation levels in CSCC.

### TRPV1 is involved in the ferroptosis process in CSCC

**FIGURE 10:**
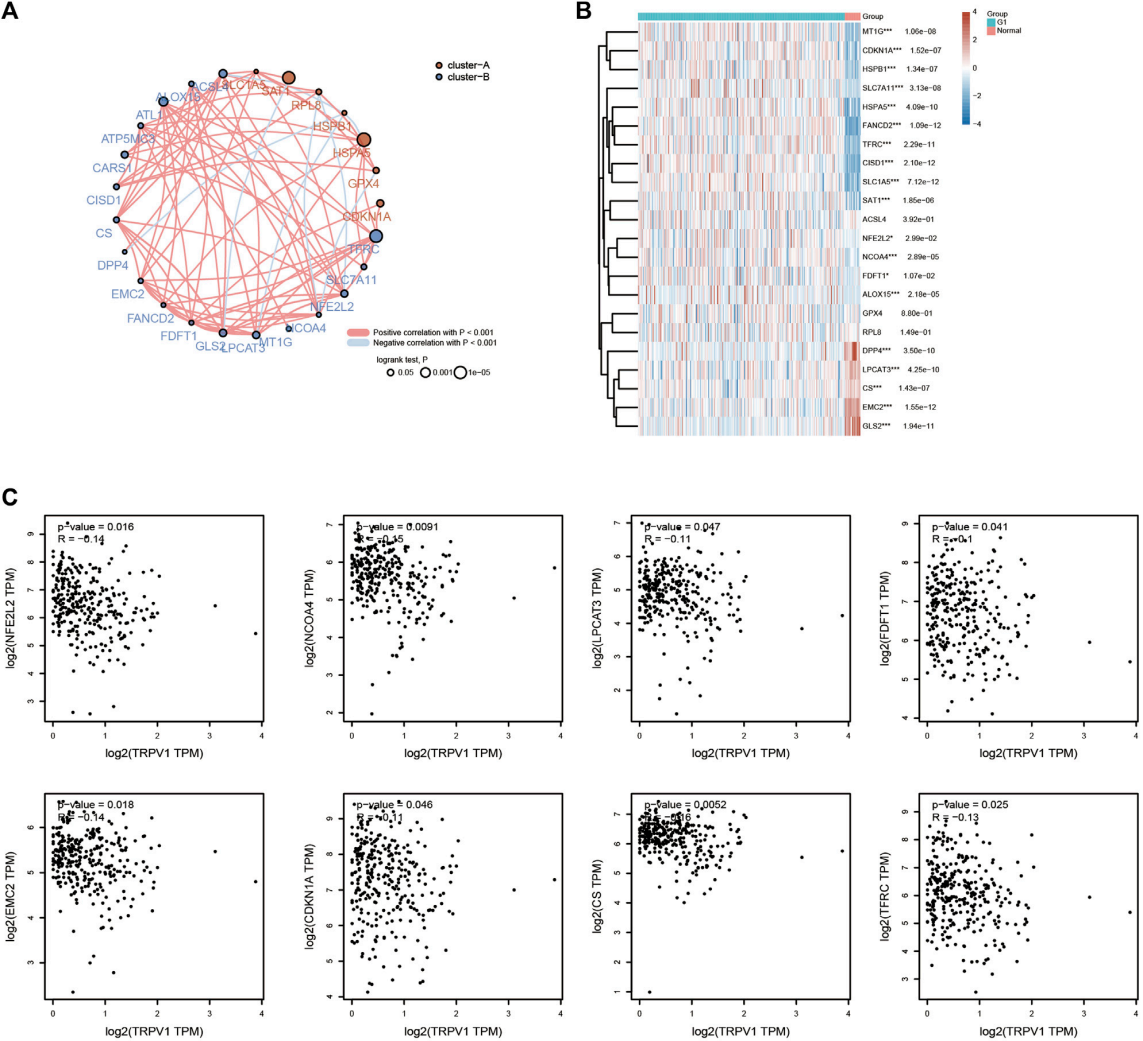
Effects of TRPV1 on ferroptosis in CSCC. **(A)** Correlation: Circles represent the ferroptosis-related genes, line represents the relationship between the ferroptosis-related genes. **(B)** Heatmap: the heatmap of ferroptosis related gene expression. **(C)** Effects of TRPV1 on the ferroptosis-related genes including NFE2L2, NCOA4, LPCAT3, FDFT1, EMC2, CDKN1A, CS, and TFRC.

GO and KEGG analysis showed low expressed TRPs affected the ferroptosis and sequestering of metal ion in CSCC. Especially, low expressed TRPV1 led to poor prognosis. It is known that ferroptosis is associated with various metabolic disorders such as iron metabolism, lipid metabolism, and antioxidant metabolism. Analysis of ferroptosis-related genes found most of the genes were positive correlation with others, and some genes presented negative correlation in CSCC (Figure 10A). Next, heatmap showed the differential expression analysis of ferroptosis-related genes (Figure 10B). TRPV1 also significantly negatively associated with NFE2L2, NCOA4, LPCAT3, FDFT1, EMC2, CDKN1A, CS, and TFRC (Figure 10C). It is reported that the function of TRPM channels is vital for cell proliferation, cell development and cell death (pyroptosis, necroptosis, and ferroptosis). As upstream signaling regulators of cell death, TRPM channels have been involved in-relevant pathologies (Shi et al., 2021). Activation of TRPV4 induces exocytosis and ferroptosis in human melanoma cells (Li et al., 2022).

### The expression pattern of TRPV1 in CSCC tissues and normal tissues

To prevent potential dataset shift, we also revisited another a publicly available database, GEO, covering cervical squamous cell carcinoma (*n* = 253), and normal patients (*n* = 19). In consistent with our previously findings, the expression of TRPV1 was significantly reduced in CSCC group compared with normal group.

**FIGURE 11:**
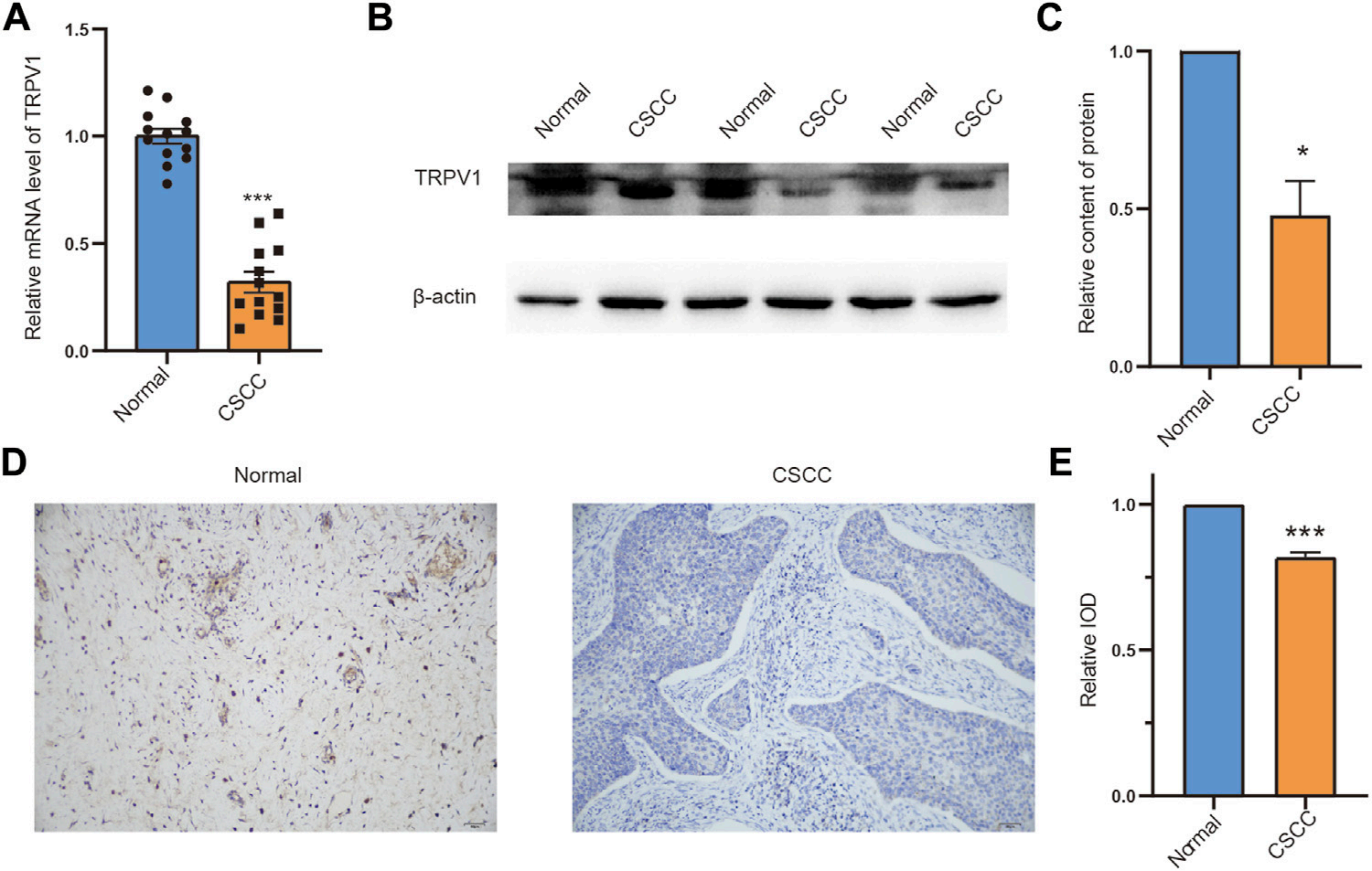
The expression pattern of TRPV1 in CSCC tissues and normal tissues **(A)** TRPV1 mRNA expression was detected by RT-qPCR. **(B,C)** Detecting protein expression levels of TRPV1 in normal tissues and CSCC tissues with western blotting. **(D,E)** Detection of the distribution of expression of TRPV1 in normal tissues and CSCC tissues by using immunohistochemical experiment. The Bar value represents the mean of three independent experiments ± SD. Compared to the normal group, *p < 0.05, ***p < 0.001.

Our study found that TRPV1 is associated with the survival prognosis of CSCC. We then determined the expression and distribution of TRPV1 in normal tissues and CSCC tissues by RT-qPCR, western blotting, and immunohistochemistry, respectively. Compared with the normal group, the expression of TRPV1 in CSCC tissue was significantly reduced (Figures 11A–C). TRPV1 is mainly distributed in the cell membrane and cytoplasm of normal tissues, however, in consistent with our previously findings, the distribution of TRPV1 in CSCC tissues was significantly reduced (Figures 11D,E). Therefore, compared with normal tissues, the expression of TRPV1 was significantly reduced in CSCC tissues.

## Discussion

TRP family members are reported to play an important role in many diseases. Here, we show that TRPV1, as a tumor suppressor gene, regulates the occurrence and development of CSCC by regulating inflammatory response, ferroptosis, and methylation levels.

We have discovered that TRPV1 is low expressed in CSCC and cervical adenocarcinoma compared to normal group, meanwhile TRPV1 showed the lower expression level in CSCC compared to cervical adenocarcinoma group. Due to the small number of cervical adenocarcinoma samples (*n* = 53), CSCC data form TCGA were analyzed for further research. Mutation analysis of TRPV1 mainly focuses on the deep deletion of TRPV1 in CSCC patients. The poor prognosis of low expressed TRPV1 is reflected in OS and DSS of CSCC patients. This shows the importance of TRPV1 in CSCC. GO and KEGG functional analysis found that TRPV1 mainly regulated inflammatory response, angiogenesis, cell differentiation, cell death (ferroptosis) and other pathways. TRPV1 mainly regulates the role of T cells and M2 macrophages, which in turn affects the occurrence and development of CSCC. Growing evidence suggests that certain TRP channels are functionally expressed in the immune system. TRPs play pivotal roles in regulating important functions of macrophages, such as cytokine and chemokine production, migration, proliferation, phagocytosis and others. TRPM7 was reported to regulate proliferation and polarisation of macrophages by stimulating IL4 secretion (Schilling et al., 2014). TRPM2 promotes the activation and cytokine production by macrophages and T cells. TRPV2^−/−^ mice show increased susceptibility to *L. monocytogenes* to regulate chemotaxis and phagocytosis by macrophages (Feske et al., 2015). At present, there are few studies on the relationship between TRPV1 and inflammation. Our research analysis can provide a new perspective for the treatment of CSCC.

TRPV1 was found to play an important role in CSCC by the methylation levels and ferroptosis. Low expressed TRPV1 promoted the methylases including YTHDF1/2/3, HNRNPA2B1, YTHDC1/2, ZC3H13, and METTL14 in CSCC. The m6A reader YTHDF1 was proved to promote ovarian cancer progression *via* augmenting EIF3C translation (Liu et al., 2020). YTHDF1 was also related to the progression of cervical cancer, non-small cell lung cancer, colorectal cancer, and gastric carcinogenesis (Shi et al., 2019; Wang et al., 2021a; Pi et al., 2021; Wang et al., 2022). YTHDF1 was considered as a potential cancer biomarker for prognosis and immunotherapy. YTHDF2 interference suppresses the EMT of cervical cancer cells (Wu et al., 2022). ZC3H13 and YTHDC1 could act as an prognostic indicator in cervical cancer (Pan et al., 2020). YTH domain containing 2 (YTHDC2) is the largest N6-Methyladenosine binding protein of the YTH protein family and the only member containing ATP-dependent RNA helicase activity. YTHDC2 which plays a significant role in epigenetic modification and immune infiltration could cause a different prognosis in cervical cancer (Zhang et al., 2021). We speculated that low expressed TRPV1 promotes cervical cancer tumorigenesis through YTHDF1/2/3, HNRNPA2B1, YTHDC1/2, ZC3H13, and METTL14 modification. Low expressed TRPV1 was also found to upregulated the ferroptosis process related genes including NFE2L2, NCOA4, LPCAT3, FDFT1, EMC2, CDKN1A, CS, and TFRC. Ferroptosis, a newly discovered iron-dependent form of cell death, contributes to various pathologies. The research on TRPV1 and ferroptosis-related genes is rarefaction in CSCC. CDC25A inhibits autophagy-mediated ferroptosis by upregulating ErbB2 through PKM2 dephosphorylation in cervical cancer cells (Wang et al., 2021b). Ferroptosis-related genes was proved to identify tumor immune microenvironment characterization for the prediction of prognosis in cervical cancer (Yang et al., 2022). The interaction between ferroptosis and immunity in the development of CC provides new insight into the molecular mechanisms of CC.

At present, TRPV1 is one of the most well studied TRP channel. After TRPV1 is activated, transient Ca^2+^ influx is induced and intracellular calcium concentration is increased, thus a series of pathophysiological changes are brought into play. In recent years, it has been found that TRP is closely related to the proliferation, invasion and metastasis of GBM, prostate cancer, liver cancer, breast cancer, bladder cancer and ovarian cancer. Our research on TRPs will form a new perspective in the treatment of CSCC disease.

## Data Availability

The original contributions presented in the study are included in the article/Supplementary Material, further inquiries can be directed to the corresponding authors.
