# Supplementary figures and images for: Role of TRPV1 ion channel in cervical squamous cell carcinoma genesis
Source: Front Mol Biosci. 2022 Aug 22;9:980262. doi: 10.3389/fmolb.2022.980262 (PMC9444153; doi:10.3389/fmolb.2022.980262)

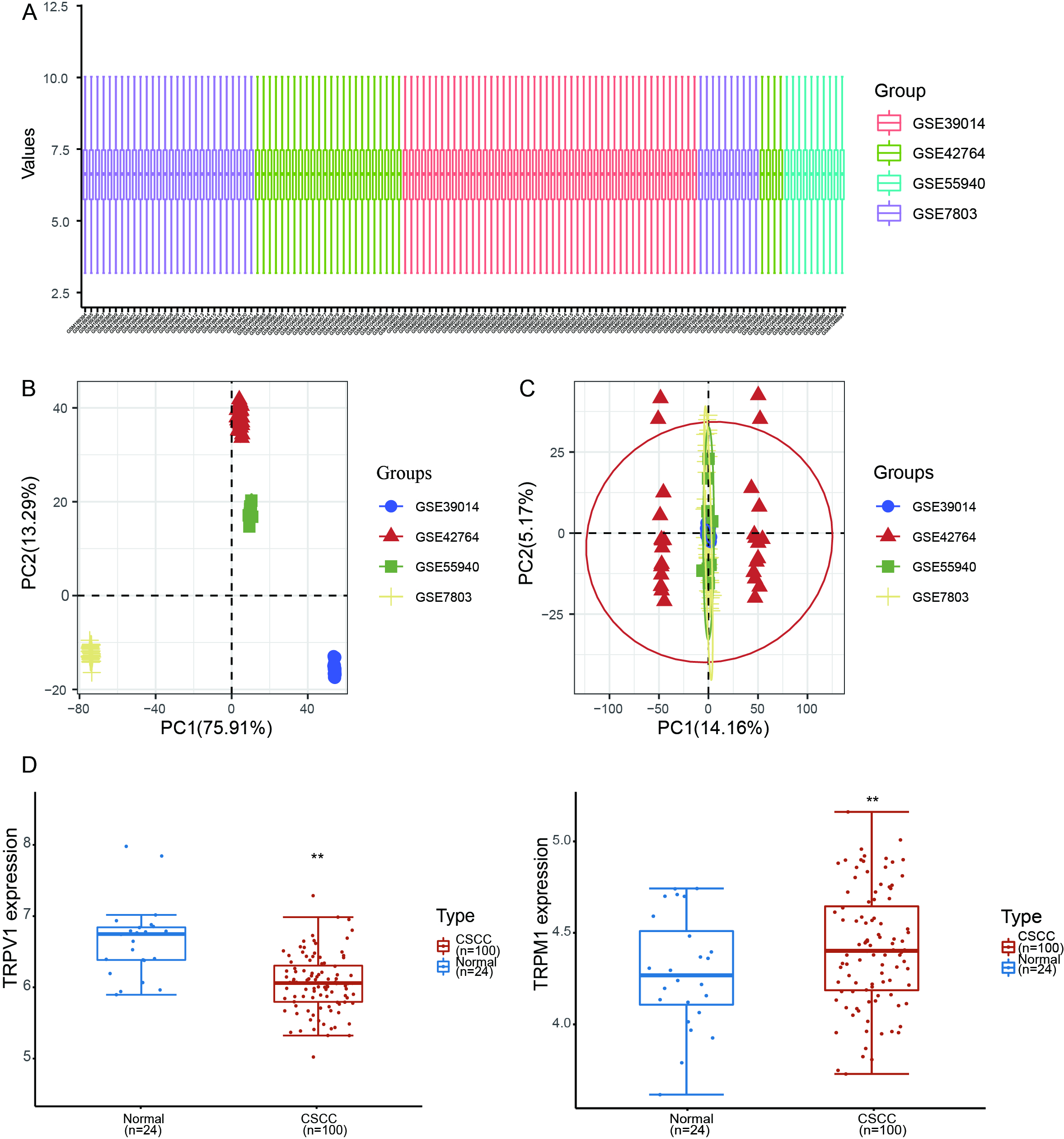

Supplement: Supplementary file 1 [file Image1.jpeg]

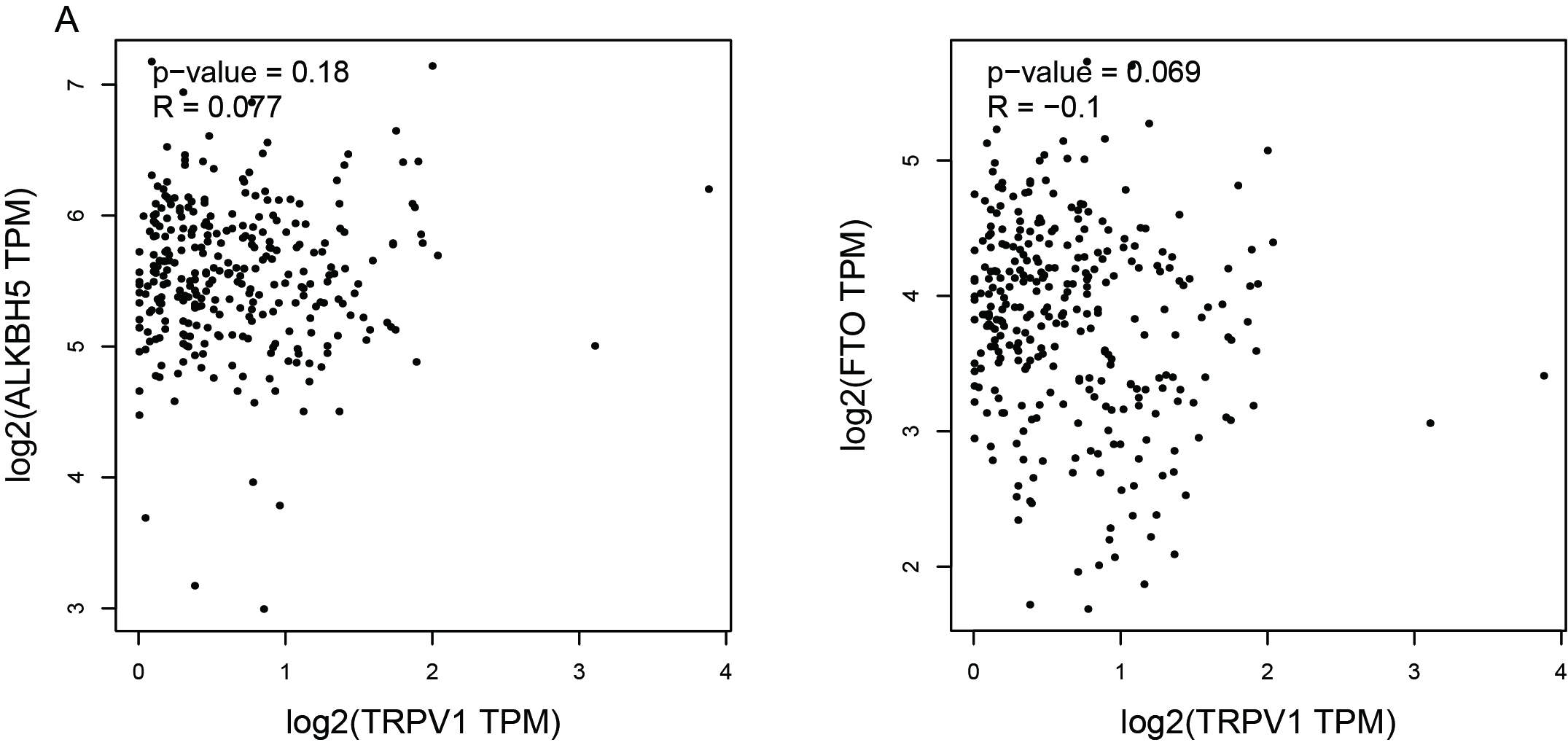

Supplement: Supplementary file 2 [file Image2.jpeg]
